# Automated measurement of alpha angle on 3D-magnetic resonance imaging in femoroacetabular impingement hips: a pilot study

**DOI:** 10.1186/s13018-022-03256-5

**Published:** 2022-07-30

**Authors:** Nastassja Pamela Ewertowski, Christoph Schleich, Daniel Benjamin Abrar, Harish S. Hosalkar, Bernd Bittersohl

**Affiliations:** 1 Department for Orthopedics and Trauma Surgery, Medical Faculty and University Hospital Düsseldorf, Heinrich-Heine-University, Düsseldorf, Germany; 2 MVZ Radiology Network NRW, Düsseldorf, Germany; 3 Department of Diagnostic and Interventional Radiology, Medical Faculty and University Hospital Düsseldorf, Heinrich-Heine-University, Düsseldorf, Germany; 4 Paradise Valley Hospital, San Diego, CA USA; 5 Tri-City Medical Center, Oceanside, CA USA; 6 Sharp Grossmont Hospital, La Mesa, CA USA; 7 Scripps Hospital, San Diego, CA USA

**Keywords:** Hip, Automatic diagnostic tool, MRI, FAI syndrome, Alpha angle, Automatic measurement

## Abstract

**Background:**

Femoroacetabular impingement (FAI) syndrome is an established pre-osteoarthritic condition. Diagnosis is based on both clinical and radiographic parameters. An abnormal manually calculated alpha angle in magnetic resonance imaging (MRI) is traditionally utilized to diagnose abnormal femoral head-neck offset. This pilot study aimed to assess the feasibility of automated alpha angle measurements in patients with FAI syndrome, and to compare automated with manual measurements data with regard to the time and effort needed in each method.

**Methods:**

Alpha angles were measured with manual and automated techniques, using postprocessing software in nineteen hip MRIs of FAI syndrome patients. Two observers conducted manual measurements. Intra- and inter-observer reproducibility and correlation of manual and automated alpha angle measurements were calculated using intra-class correlation (ICC) analysis. Both techniques were compared regarding the time taken (in minutes) and effort required, measured as the amount of mouse button presses performed.

**Results:**

The first observer’s intra-observer reproducibility was good (ICC 0.77; p < 0.001) while the second observer’s was good-to-excellent (ICC 0.93; p < 0.001). Inter-observer reproducibility between both observers in the first (ICC 0.45; p < 0.001) and second (ICC 0.56; p < 0.001) manual alpha angle assessment was moderate. The intra-class correlation coefficients between manual and automated alpha angle measurements were ICC = 0.24 (p = 0.052; observer 1, 1st measurement), ICC = 0.32 (p = 0.015; observer 1, 2nd measurement), ICC = 0.50 (p < 0.001; observer 2, 1st measurement), and ICC = 0.45 (p < 0.001; observer 2, 2nd measurement). Average runtime for automatic processing of the image data for the automated assessment was 16.6 ± 1.9 min. Automatic alpha angle measurements took longer (time difference: 14.6 ± 3.9 min; p < 0.001) but required less effort (difference in button presses: 231 ± 23; p < 0.001). While the automatic processing is running, the user can perform other tasks.

**Conclusions:**

This pilot study demonstrates that objective and reliable automated alpha angle measurement of MRIs in FAI syndrome hips is feasible.

*Trial registration* The Ethics Committee of the University of Düsseldorf approved our study (Registry-ID: 2017084398).

## Background

Femoroacetabular impingement (FAI) syndrome is a pathological condition of the hip joint leading to premature osteoarthritis (OA) [1]. FAI involves a dynamic conflict between the femoral head and acetabulum arising from abnormal morphology. Based on the part of the hip involved, the FAI syndrome is subclassified into: Cam-type, characterized by the femoral head’s asphericity, based on a bone excess on the femoral head or head-neck junction [2, 3], and the Pincer-type, with increased acetabular coverage of the femoral head (focal or general). Often the presentation is mixed [4, 5].

Early intervention and management play a key role in modifying the natural history of the FAI syndrome, potentially preventing progressive cartilage damage leading to early osteoarthritis. Therefore, early diagnosis with reliable and reproducible imaging features remains important [6–8].

Diagnosis is based on clinical and radiographic parameters, including a detailed history, physical examination findings, and radiological parameters [2]. Plain radiographs are utilized to assess bony morphologies, while magnetic resonance imaging (MRI) enables evaluation of the cartilage status [9]. Alpha angles have been used to quantify the concavity (head-neck offset) of the femoral head-neck junction [10–12]. These alpha angles can be obtained with conventional radiographic imaging for FAI compromising two radiographs: an anteroposterior pelvic view and a lateral view such as an axial cross-table view of the proximal femur, the Lauenstein- or Dunn view. However, morphological evaluation on conventional radiography allows for a two-dimensional (2D) assessment only and, hence, may be limited in establishing a valid hip joint status in FAI. This concern has been outlined by Dudda et al., who noted pathologically increased alpha-angles even in hip joints with normal-appearing radiographs [13]. Therefore, despite being advantageous and less cumbersome in clinical practice, FAI syndrome prediction based on alpha angle assessment in biplanar radiographs has its limitations. Consequently, alpha angle measurements are often taken in radial MRI cuts to assess the hip-neck intersection around the femoral head. However, manual generation of radial MRIs and alpha angle construction and calculation is quite time-consuming. During manual construction and calculation of the alpha angle, the observer must define landmarks and measure distances and angles by hand while utilizing standard radiologic software. Therefore, the alpha angle measurement may vary between measurements (intra-observer reproducibility) and between observers (inter-observer reproducibility).

A reliable and accurate alpha angle measurement in FAI syndrome diagnosis remains a challenge. Given the advancements in automated diagnostic tools for image interpretation, automated alpha angle measurements may hold promise for the objective, observer-independent, reliable, and potentially effortless diagnosis of the FAI syndrome. Ettinger et al. [14] have described an automated image interpretation technique for knee arthroplasty, which is known to improve the surgical outcome. More meticulous preoperative planning and intra-operative guidance to the correct implant placement and positioning are possible with automated image interpretation [14]. Another example of a promising automated diagnostic tool in orthopedics was described by Hesper et al. [15], who reported the automated use of a 3D delayed gadolinium-enhanced MRI of cartilage (dGEMRIC) technique (sensitive to early loss of cartilage glycosaminoglycan in the course of cartilage degeneration) [16–18]. Their findings are similar to those of Schmaranzer et al., who compared a 3D analysis of cartilage thickness and dGEMRIC index using both a manual and a new automated method [19]. They noted an accurate, reliable, and reproducible analysis of dGEMRIC indices, thickness, surface area, and volume using the automatic segmentation of hip cartilage.

In this present pilot study, we sought to evaluate the feasibility of automated alpha angle measurements in radial MRIs. The primary objective of this study was to investigate differences (if any) between alpha angle measurements obtained with manual and automated methods in FAI syndrome hip MRIs. Secondary objectives were (1) to assess inter- and intra-observer reproducibility in the manually performed measurements; (2) to evaluate correlation of automated data with manual data; and (3) to identify any differences between the assessing time (measured in minutes) and effort (based on the amount of mouse button presses) for both measurement types. To this end, we compared alpha angle measurements obtained with both manual and automated methods in FAI syndrome hip MRIs. We hypothesized that automated alpha angle measurements in radial MRIs correlate with manual data, which incurs less effort and time than manual alpha angle measurements.

## Methods

### Study population

Following the local ethics committee approval, we performed a comparative, retrospective feasibility study on MRI data of FAI syndrome patients who underwent hip arthroscopy at our institution (Department of Orthopedics at Medical Faculty of University of Düsseldorf, Germany) between 2010 and 2018. The inclusion criteria were clinically and arthroscopically confirmed FAI syndrome diagnosis, availability of a good-quality MRI data set of the hip, and a 3D double-echo steady-state sequence (DESS) with high-resolution, undistorted images deprived of artifacts, and good visualization of anatomic structures. The diagnosis of FAI syndrome was not based on a single clinical sign or radiological finding. Instead, the diagnosis of FAI syndrome was verified by a triad of symptoms, clinical signs, radiological and intraoperative observations including motion-related or position-related pain in the hip, clicking symptoms, limited range of motion in particular flexion and internal rotation, a positive flexion-adduction-internal rotation (FADIR) test, a reduced femoral head-neck offset and characteristic associated cartilage and labral lesions within the region of impingement. A total of 52 MRI data sets (52 patients, 34 males, 18 females) were screened from the institutional database and subsequently contacted. Of these, 32 patients were excluded because of the absence of written informed consent for our retrospective study. One other patient had to be excluded from our investigations as the MR scanner was different to that used to obtain the rest of the data. Subsequently, a total of 19 patients (ten males, nine females, mean age: 31.5 ± 12.4 years, age range: 11–57 years, nine right hips, ten left hips) were included in our preliminary study. All patients gave written informed consent to use their anonymized data.

### Magnetic resonance imaging

MRI was conducted using a 3 T scanner (MAGNETOM Trio, Siemens Healthcare, Erlangen, Germany) with a 4-channel phased-array coil. Patients were in the supine position while the examined leg was stabilized with textiles to prevent motion artifacts and increase comfort during examination. The MR sequences included: (1) localizer images, (2) standard pulse sequences, and (3) a high-resolution 3D double-echo steady-state (DESS) sequence for morphological cartilage assessment (TR 14.8 ms, TE 5.03 ms, flip angle 25°, NEX 1, FOV 192 mm^2^, slice thickness 0.6 mm, in-plane resolution 0.6 × 0.6 mm, bandwidth 260 Hz/pixel, acquisition time 13.17 min).

### Manual radial image reformating

The 3D DESS data sets were transferred to a Leonardo stand-alone workplace (Siemens Healthcare GmbH, Erlangen, Germany). Based on previously published studies, a multiplanar reconstruction software was used to create seven radial reformats through the femoral head [20]. Radial cuts were arranged concentrically toward the femoral head and perpendicularly arranged toward the acetabulum: (1) anterior, (2) anterior–superior, (3) superior-anterior, (4) superior, (5) superior-posterior, (6) posterior-superior, and (7) posterior [21]. The time and effort of the manual development of seven radial slices were assessed. The untrained observer (observer 1) was instructed by the expert observer (observer 2) in generating radial slices. Observer 1 reformatted seven radial portions in each hip MRI twice for the purpose of training. These results were not included in the actual analysis. Observer 1 then generated seven radial slices in each hip MRI twice (measurement 1 and 2) during a total period of four weeks. For each measurement, time spent (in minutes) and the effort (counted amount of used mouse button presses) taken to generate the measurement was documented. Time and clicks were counted manually.

### Manual alpha angle measurement

**Fig. 1:**
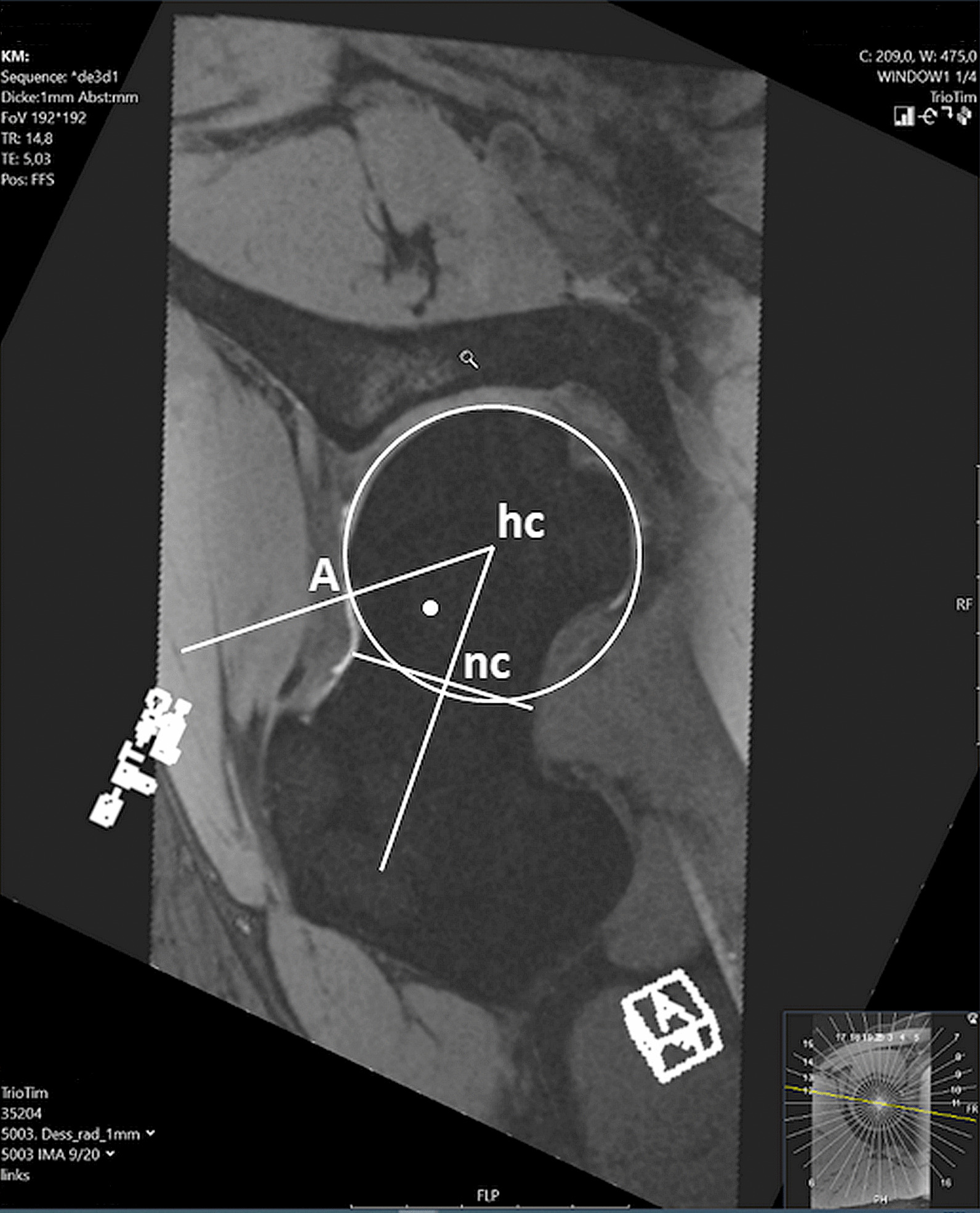
Manual alpha angle measurement. ‘hc’ = center of the femoral head. ‘nc’ = center of the femoral neck located at the neck's most narrowed point. ‘A’ = point where the distance from the bone to the center of the femoral head exceeds the radius of the best-fit circle around the femoral head

In this study, alpha angles were manually measured using the method described by Nötzli et al. [11]. First, a "circle of best fit," a circle best fitting the anatomic femoral head's roundness, was constructed. The center of the head was labeled ‘hc’. Secondly, the center of the neck (nc) was identified at the neck's most narrowed point. The points nc and hc were connected through a line. Point A was defined, representing the extent of the femoral neck's concavity, or the point where the distance from the bone to the center of the head (hc) first exceeds the radius. The alpha angle was then measured between ‘A-hc’ and ‘hc-nc’ in the seven radial cuts of all nineteen hips (Fig. 1).

The manual alpha angle measurements of all hips were taken by a) an initially inexperienced observer 1 (Ph.D. student after completing her medical studies), and b) an expert observer 2, who was highly experienced (over 15 years) in musculoskeletal imaging. Observer 2 instructed the untrained observer 1 in assessing the alpha angle. For training, Observer 1 performed the alpha angle measurements in the seven radial planes in all nineteen hip MRIs twice. These results were not included in the actual analysis. Subsequently, observer 1 repeated the manual alpha angle measurement the first time for analysis (measurement 1). Besides the alpha angles, a particular focus was placed on the time spent (in minutes) and the effort (number of mouse button presses) it took to perform measurements. Again, time and clicks were counted manually. For the intra-observer reproducibility assessment, observer 1 repeated the analyses (measurement 2) four weeks after measurement 1. The time and effort spent performing measurement 2 were documented. For inter-observer reproducibility, observer 2 independently carried out manual alpha angle measurements (measurements 3 and 4) analogously to measurements 1 and 2. All measurements were taken separately, and there was a four-week interval between the first and the second measurements to eliminate the memory effect. Notably, the observers measured the alpha angles strictly according to the method described by Nötzli et al. [11], were aware that the time and number of clicks were recorded, and were tutored to prioritize precession over time when performing their calculations.

### Automated alpha angle measurement

The 3D DESS data were transmitted to a "standalone" workplace (DESKTOP-P810HEA, processor: Intel® Core™ i7-7700 CPU @ 3.60 GHz, RAM: 16 G.B., System type: 64-Bit-operating system, Windows 10 Pro, no professional medical product). The automated computer-based prototype software (MR Chondral Health 2.1, Siemens Healthcare GmbH, Erlangen, Germany) measured alpha angles in all nineteen hips (measurement 5). The software targets cartilage segmentation and generates alpha angles as an additional result. Every software analysis of each hip was estimated in time (minutes) and the total number of mouse button clicks/presses. The time taken for uploading the image data to the computer was documented separately.

A fully automated approach was used to calculate alpha angles throughout the hip, which was made available as a single step in the workflow of the prototype software. The procedure for determining the alpha angle is based on the study by Xia et al. [22]. However, the implementation differs in detail, especially when choosing the radial slice group, and was realized with other components. The algorithm is described in detail by Xia et al. [22]. The main steps are summarized as follows:

1. Bone segmentation: a segmentation mask that marks the pixels belonging to the hip bones is the input taken by the alpha angle algorithm. The bone segmentation is calculated as an internal step in the MR Chondral Health prototype and is based on an approach that uses Active Shape Models [23]. The bone segmentation is performed on a high-resolution 3D MR image of suitable contrast (intermediate weighted and with a suppressed fat signal; in this work, a DESS).
2. 3D femur bone surface: from the binary mask of the bone segmentation, the surface of the femur bone is defined and generated from corner points, edges and triangles.
3. Landmark detection: using a landmark detection based on the ALPHA framework, the center of the femoral head and the femur neck axis are first approximately determined [24].
4. Determination of the surface of the femoral head and the femur neck: in the next step, the surface of the femoral head and the surface of the femur neck are determined using the position of the individual corner points relative to the femoral head center and femur neck within the entire surface of the femur bone.
5. More precise determination of the femoral neck and femoral head center: with the help of a sphere fitting using the Ransac algorithm, the center of the femoral head and its radius can now be determined more precisely. The circle with the smallest circumference is specified on the surface of the femur neck, which then defines a plane. The normal to this plane through the center of the femoral head provides a more precise estimate of the axis of the hip neck. A radial layer group is determined by this more precisely defined axis of the femur neck.
6. Alpha angle determination: the alpha angles are determined slice-by-slice on this slice group. For this purpose, a 2D map is created that describes the roundness of the femoral head in spherical coordinates at the respective position.
7. The alpha angles are then calculated on this map, depending on the coordinates on the map, as an optimal horizontal cut, which corresponds to the minimization of a cost function. The version of the cost function used in MR Chondral Health 2.1 differs in the smoothness condition so that it allows a little more flexibility, but this does not make any conceptual difference. Further details can be found elsewhere [22].

**Fig. 2:**
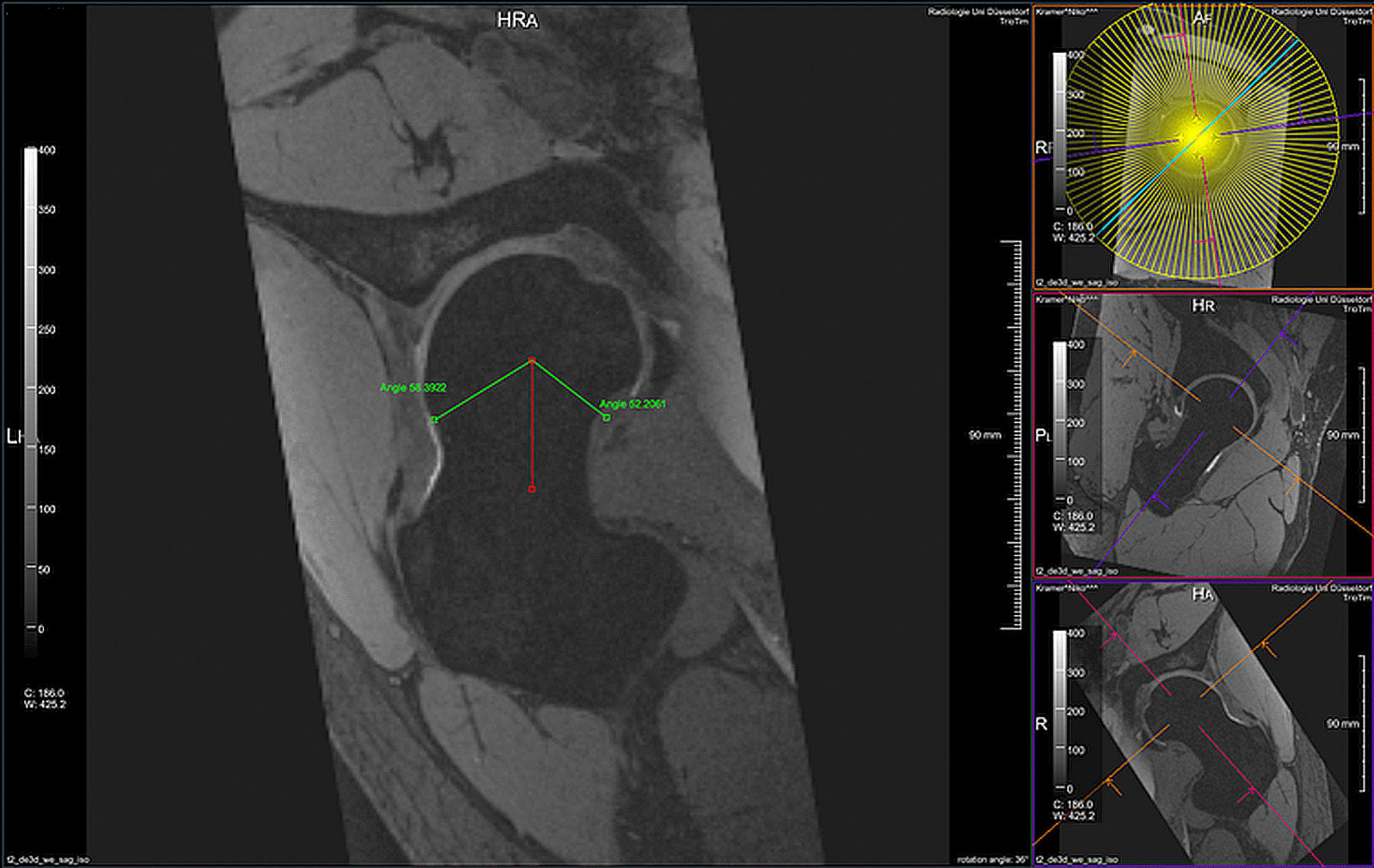
Automated alpha angle measurement using automated computer-based prototype software (MR Chondral Health 2.1, Siemens Healthcare GmbH, Erlangen, Germany)

Notably, the software can measure alpha angles in up to 120 radial images (Fig. 2). We selected those planes that best fitted the seven manually derived radial sections to facilitate subsequent correlation of automated alpha angle assessment with alpha angle measurement.

### Statistics

IBM SPSS Statistics for Macintosh (Version 28.0, Armonk NY: IBM Corp.) was used for statistical analyses. Values were presented as mean ± standard deviation (SD). P values below 0.05 were considered statistically significant. Intra- and inter-observer agreement between the manual measurements and the manual and automated alpha angle measurement correlation were assessed using the intra-class correlation coefficient (ICC) for absolute agreement. For the ICC assessments, a 95% confidence interval was assessed and reported in square brackets ([…]). ICC values > 0.9 were considered “excellent reliability”. Values < 0.9 and ≥ 0.75 indicated good reliability, whereas values < 0.75 and ≥ 0.5 showed moderate reliability, and values < 0.5 showed poor agreement [25]. The Student’s paired t test was used to compare mean alpha angles, the required time, and clicks between the manual and automated alpha angle measurements. A Bland–Altman analysis was performed to display the relationship between the manual and the computerized alpha angle measurement. The blandr r package was used to create the Bland–Altman plots [26].

## Results

The following results are summarized in Table 1. The mean manual alpha angle measured by observer 1 was 61 ± 19°, with the range being 32°–127°. The mean manual alpha angle measured by observer 2 was 54 ± 14° (range: 29°–108°). The mean automated alpha angle was 57 ± 6° (range: 44°–72°). The differences between these measurements were statistically different: p < 0.001 (observer 1 versus observer 2), p = 0.008 (observer 1 versus automated alpha angle measurement), and p = 0.014 (observer 2 versus automated alpha angle measurement).

**Table 1:** Alpha angle values in seven radial MRI planes of 19 FAI (femoroacetabular impingement) hip joints (= 133 measurements in total) including mean, standard deviation (SD), minimum, maximum and percentile values, obtained with manual and automated software assessments

	**Mean**	**SD**	**Min**	**Max**	**Percentiles**		
					**25**	**50**	**75**
**Observer 1** Measurement 1	61.38	19.04	32.00	127.00	46.35	57.80	73.35
**Observer 1** Measurement 2	58.57	18.40	29.00	112.00	42.80	53.40	70.70
**Observer 2** Measurement 3	54.36	14.30	29.00	108.00	43.50	52.00	62.50
**Observer 2** Measurement 4	52.61	13.70	29.00	105.00	43.00	50.00	60.00
**Software** Measurement 5	57.07	5.80	44.00	72.00	52.50	56.00	62.00

**Fig. 3:**
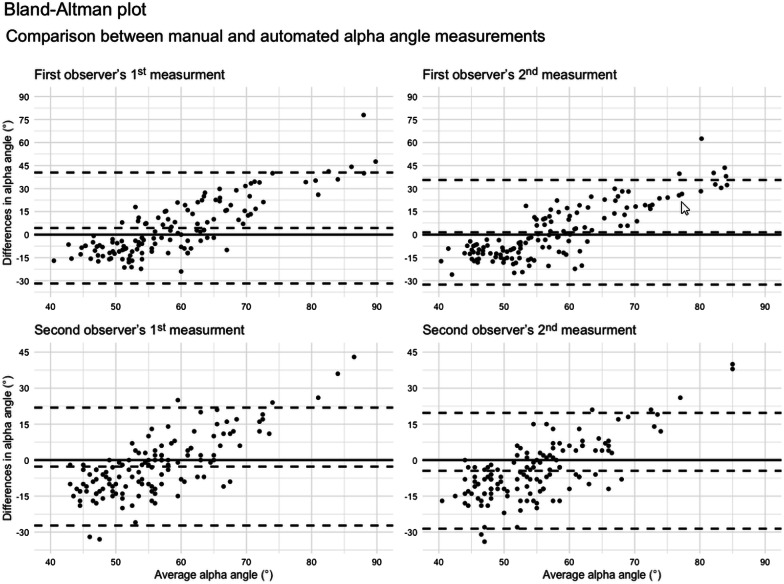
Bland–Altman plot comparison between manual and automated alpha angle measurement. All alpha angle measurements in the seven radial imaging planes were considered in the evaluations. The x-axis displays the average alpha angle of the two measurement techniques. The y-axis illustrates the difference in the alpha angle measurements between the two measurement instruments (manual–automated). The three dashed lines display the average difference in alpha angles, the upper and the lower limit of agreement for the average difference between the manual and automated alpha angle measurement. The limits of agreement spread were considerably larger for observer 1 (1st—31.8–40.5; 2nd—32.6–35.6) than for observer 2 (1st—27.3–21.9; 2nd—28.6–19.7)

The Bland–Altman plot shows that the range of deviations between the manual and automated alpha angle measurements was more extensive for observer 1 than for observer 2 (Fig. 3).

ICC for observer 1’s two alpha-angle measurements was 0.77 [0.68–0.84], p < 0.001. ICC for observers 2’s two alpha-angle measurements was 0.93 [0.90–0.95], p < 0.001. Analysis of inter-observer reproducibility revealed an ICC of 0.45 [0.22–0.61], p < 0.001.

The ICC between observer 1’s first manual alpha angle measurement and the automatically assessed alpha angle was 0.24 [-0.06–0.45], p = 0.052. ICC between observer 1’s second measured alpha angle and the automatically measured alpha angle was 0.32 [0.04–0.52], p = 0.015. Between observer 2’s manually measured alpha angles and the automatically measured alpha angle, the ICC values were 0.50 [0.30–0.64], p < 0.001 and 0.45 [0.22–0.61], p < 0.001.

The following results are summarized in Table 2. The mean time of observer 1’s first measurement of alpha angle was 24.5 ± 6.3 min. The mean time for generating radial images in the first manual measurement by observer 1 was 0.9 ± 0.2 min. The total mean time for the manual alpha angle measurement 1 and manual generation of radial images was 25.4 ± 6.3 min. The mean time for observer 1’s second manual measurement of alpha angle decreased to 10.5 ± 2.8 min. The mean time for generating radial images in the observers’ second measurement was 0.8 ± 0.1 min. The total mean time for alpha angle measurement 2 and radial image generation was 11.2 ± 2.7 min. The time difference between the first and second measurements, including radial image generating, was 14.2 ± 5.1 min (p < 0.001).

**Table 2:** Assessment of time (measured in minutes) and effort (measured in mouse button presses/clicks) of observer 1 in seven radial MRI planes of 19 FAI (femoroacetabular impingement) hip joints, including mean ± standard deviation, using either the manual or the automated software method, and P-values demonstrating differences in time and clicks between the manual measurement 1 and 2 and between both manual measurements and the automated measurement

			Mean	P-value
**Manual reformating** Seven radial MRI planes	Time (min)	Measurement 1	0.9 ± 0.2	0.003
		Measurement 2	0.8 ± 0.1	
	Clicks	Measurement 1	18.8 ± 2.8	0.263
		Measurement 2	18.1 ± 1.9	
**Manual calculation** Alpha angles	Time (min)	Measurement 1	24.5 ± 6.3	< 0.001
		Measurement 2	10.5 ± 2.8	
	Clicks	Measurement 1	1113.9 ± 95.1	< 0.001
		Measurement 2	235.2 ± 23.8	
**Manual total** Reformating + Calculation	Time (min)	Measurement 1	25.4 ± 6.3	< 0.001
		Measurement 2	11.2 ± 2.7	
	Clicks	Measurement 1	1132.7 ± 95.1	< 0.001
		Measurement 2	253.3 ± 23.8	
**Software**	Time (min)	CD Uploading	16.6 ± 1.9	
	Time (min)	Measurement 5	25.8 ± 2.8	< 0.001
	Clicks	Measurement 5	22.8 ± 3.3	< 0.001

The mean amount of mouse button presses in observer 1’s first measurement of alpha angles was 1114 ± 95, and the mean amount of mouse button presses for generating radial images in the first measurement by observer 1 was 19 ± 3; therefore, the total mean amount of mouse button presses in the first manual measurement of observer 1 was 1133 ± 95. The mean amount of mouse button presses in observer 1’s second measurement of alpha angles decreased to 235 ± 24, and the mean amount of mouse button presses for generating radial images in observer 1’s second measurement was 18 ± 2. The total mean amount of mouse button presses for alpha angle measurement and generating radial images in the second manual measurement (observer 1) was 253 ± 24. The difference in the total mean amount of mouse button presses between the first and second measurements, including radial image reformatting, was 879 ± 110, p < 0.001.

The mean time for automatic alpha angle measurement was 25.8 ± 2.8 min. The mean time for uploading image data for the automated assessment was 16.6 ± 1.9 min. The mean amount of mouse button presses for the automatic alpha angle measurement was 23 ± 3.

The time difference between the first manual measurement (observer 1) and the automatic measurement, including radial image generation and alpha angle measurement, was 0.4 ± 6.2 min, p = 0.768. The difference between the mean amount of mouse button presses in the first manual measurement (observer 1) and the automatic alpha angle measurement generation, including radial image generation and alpha angle assessment, was 1110 ± 97, p < 0.001.

The total time difference between the second manual measurement (observer 1), including radial image generation and automatic alpha angle measurement, was − 14.6 ± 3.9 min, p < 0.001. The difference in the total mean amount of mouse button presses between the second manual measurement (observer 1) and the automatic alpha angle measurement, including radial image generation and alpha angle measurement, was 231 ± 23, p < 0.001.

## Discussion

Previously published studies have shown that automated diagnostic tools in health care can save time and that diagnostics can, as a result, be achieved in a more detailed and reproducible manner [14, 15, 27]. Because early diagnosis in FAI syndrome can help with early intervention (and change the course of the disease), accurate and reliable imaging-based diagnosis is a highly relevant target [2].

Anatomical abnormalities of the femoral head-neck offset change, traditionally identified with manual alpha angle measurements, are reader-dependent and potentially less reliable. This pilot study sought to determine the feasibility of an automated diagnostic tool for a reliable and objective assessment of alpha angle in FAI syndrome patients.

In this study, observer 1’s (non-expert) intra-observer reproducibility was good (ICC 0.77; p < 0.001). Observer 2’s (expert) intra-observer reproducibility can be considered excellent (ICC 0.93; p < 0.001). However, the inter-observer reproducibility between observer 1 and observer 2 in the first (ICC 0.45; p < 0.001) and second (ICC 0.56; p < 0.001) manual alpha angle measurement was poor or moderate, indicating that manual measurements are reader-dependent and may not always be reliable. In general, we noted little agreement between the manual and automated correlation; the correlations between automatically generated alpha angles and manually assessed alpha angles by observer 2 in the first (ICC = 0.50, p < 0.001) and the second (ICC = 0.45, p < 0.001) measurement were moderate or poor. The correlation between automatically generated alpha angles and manually assessed alpha angles by observer 1 did not reach statistical significance in the first (ICC = 0.24, p = 0.052) and was poor in the second (ICC = 0.32, p = 0.015) measurement. In further studies, the findings of this study need to be evaluated in more cases. On the other hand, the values obtained seem to indicate that inexperienced evaluators in particular could benefit from automated measurement.

The efforts in the manual alpha angle measurements of untrained observer 1 were high, regarding both time and the number of mouse clicks, especially in the first measurements. Through repetitive measures, the manual alpha measurements' effort by the untrained observer 1 was reduced timewise (24.5 ± 6.3 min *vs*. 10.5 ± 2.8 min; p < 0.001) and in amounts of mouse button presses (1114 ± 95 clicks *vs*. 879 ± 110 clicks; p < 0.001). The effort it took to generate alpha angles automatically was low in mouse button presses (23 ± 3) but high timewise (25.8 ± 2.8 min). The automatic measurement may require more time but provides a more detailed analysis of the hip joint (alpha angles in 120 regions). With manual alpha angle measurements in only seven radial slices, as performed in this and previous studies, pathologic morphology can potentially be underestimated. Furthermore, the upload time did not cost the observer any effort in terms of mouse clicks, which means the healthcare worker guiding the automatic measurement could use this time elsewhere—the most time-consuming part was uploading the 3D MRI data set into the software, lasting 16.6 ± 1.9 min in our setting. After uploading, the time and effort needed were also low. As computer and data processing technologies develop, we assume that upload time will play a minor role in the future. This could already be accelerated today by using a more powerful PC. In addition, it must be noted that the software also performs a cartilage segmentation over the measurement time, which is the main feature and most time-consuming part of running the software. At some point, the software could even be integrated into radiologic tools, so that uploading would not be necessary in the future, and alpha angles would be immediately generated automatically.

Previous studies have investigated automated diagnostic tools for hip diseases. But most of these studies were performed based on plain radiographic images [28–30], ultrasound images [31], or computed tomography (CT) images [32]. Fischer et al. investigated an automated morphometric analysis framework for the quantitative analysis of geometric hip joint parameters in MR images from the German National Cohort (GNC) study [33]. Their secondary analysis on 40 participants (mean age, 51 years; age range, 30–67 years; 25 women) involved a morphometric assessment, which was based on a proton density-weighted 3D fast spin-echo sequence, calculating the centrum-collum-diaphyseal, center-edge (CE), three alpha angles, head-neck offset (HNO), and HNO ratio along with the acetabular depth, inclination, and anteversion. Compared with manual assessments, high agreement in mean Dice similarity coefficients (average of 97.52% ± 0.46) with low mean median absolute deviations (MAD) values were noted. Damopoulos et al. published their study results on a system for the segmentation of the proximal femur from radial MRI scans and the reconstruction of its 3D model that may be used to diagnose and plan hip-preserving surgery [34]. Their dataset consisted of the radial MRI scans of 25 patients with FAI syndrome or AVN and accompanying manual segmentation of the femur, treated as the ground truth. They achieved a good agreement between both methods, similar to those of Fischer et al. We are also aware of other automatic diagnostic tool studies using MRI-based images to perform automated cartilage segmentation [19, 35, 36]. The results of our study indicate that automatic alpha angle measurements are feasible and may play a larger role in clinical practice going forward. We believe this pilot study demonstrates an objective and reader-independent automated alpha angle assessment. As long as uploading the MRI data sets is required, it may take an equivalent time to analyze the morphology of one hip MRI. Still, generating up to 120 alpha angle measurements per hip MRI makes the automated method more detailed and incurs less effort in mouse button clicks than the manual alpha angle measurement. Further studies should involve a more extensive and varied population, as well as different software systems tested on different stand-alone computer types. Finally, it should also be mentioned that merely a more straightforward method for classifying the FAI syndrome, e.g., using only three stages of severity in three radiographic views, may likewise enable a simplification and a higher reproducibility of the assessment [37].

This pilot study has limitations: the algorithm in this pilot study is understood to be the first attempt at an automated alpha angle determination. Hence, the evaluation of these initial results is only the beginning. A further project could investigate the technique's weaknesses, using manual measurements on various MR imaging series to further improve the algorithm with the help of the manual data. In addition, our study population with nineteen patients was relatively small, and a power analysis was not performed in this preliminary study. Studies with sufficient populations are needed to verify our findings. Another limitation was the absence of various software types for the automatic alpha angle measurements. In further studies, different software types should be utilized to guarantee software manufacturer-independent results. The automated measurements were taken in one stand-alone computer type only. In other studies, different computer types with various power settings and additional hardware, e.g., graphic cards, could be implemented to investigate their influence on the automatic measurement time. Furthermore, due to the rather gross identification of seven matching MR sections, it is possible that the automated and manually derived corresponding MRI regions were slightly different due to a mismatch in the plane orientation. In the manual measurement, the middle of the femoral neck is determined at the narrowest point in each MR image. This point, together with the femoral head center and the point at which the femoral head leaves the best-fit circle, is used to measure the alpha angle. With the computer-based measurement, a femoral neck axis is determined for the entire 3D data set; this is a potential cause of discrepancy. Another limitation was the measurement of FAI syndrome hips only. Further studies need to include alpha angle measurements in healthy control cohorts and in hips with other potential femoral head deformities, such as hip dysplasia or Legg–Calve–Perthes disease. Observer 1 was inexperienced in calculating alpha angles in radial MRI cuts. The rationale for this approach was to point out the benefit of an automatic measurement that does not require extensive training because FAI may not be an everyday diagnosis in the clinical routine. The effect is easily seen in the considerable reduction in range of deviations between the manual and automated alpha angle measurements, in time and clicks, indicating a learning effect. However, it must also be noted that this methodology could possibly have inflated the inter-rater reliability as the second observer taught the first observer on how to take the measurements. Further studies are needed to verify our findings in inter- and intra-observer reproducibility. These studies should potentially involve a higher number of trained and untrained observers. In addition, assessing the effort by counting the used mouse clicks may have affected the observer's performance. In general, the study design was simple and included basic questions. Nevertheless, by answering these questions in our pilot study, the foundation for further studies in this field has been set.

## Conclusion

This pilot study demonstrates that manual alpha angle measurements in FAI syndrome hip MRIs are reader-dependent and may not be reliable. In contrast, a fully automated objective MRI alpha angle assessment is feasible and can save healthcare workers time and effort. Further studies are necessary to substantiate our findings and enhance the software development in automatically generating alpha angles throughout the femoral head.

## Data Availability

All data and materials including this article will be available upon request via email to the first author.

## Abbreviations

MRI: Magnetic resonance imaging
FAI: Femoroacetabular Impingement
dGEMRIC: Delayed gadolinium-enhanced magnetic resonance imagines of cartilage
Cam-FAI: Cam-Femoroacetabular Impingement
Pincer-FAI: Pincer-Femoroacetabular Impingement
OA: Osteoarthritis
3D: Three-dimensional
Min: Minutes
